# Control and eradication of bovine trichomonosis in Wyoming, USA by testing and culling positive bulls

**DOI:** 10.1186/s13567-021-00996-w

**Published:** 2021-10-07

**Authors:** Chaoqun Yao

**Affiliations:** Department of Biomedical Sciences and One Health Center for Zoonoses and Tropical Veterinary Medicine, Ross University School of Veterinary Medicine, St. Kitts, P.O. Box 334, Basseterre, St. Kitts and Nevis

**Keywords:** trichomonosis, trichomoniasis, *Tritrichomonas foetus*, disease control, disease eradication

## Abstract

Bovine trichomonosis is caused by *Tritrichomonas foetus*. Thirty-three US states have state rules on this disease and render it reportable due to potential huge economic losses to cattle industry. The various rules of different states generally mandate testing and culling *T. foetus*-positive bulls as well as prohibiting import of *T. foetus*-positive animals. Wyoming has enforced these rules for over 20 year beginning in 2000. From 2017 to 2019, 3 years in a row, not even one *T. foetus*-positive bull has been detected throughout the entire state among over ten thousand bulls tested annually. Wyoming is the first US state to achieve total control and eradication of bovine trichomonosis by testing and culling *T. foetus*-positive bulls.

## Introduction

Bovine trichomonosis is a sexually transmitted disease in cattle. Its causative pathogen is *Tritrichomonas foetus*, which is closely related to a human trichomonad protozoan *Trichomonas vaginalis*. The parasite is transmitted by direct sexual contact. An infected bull may appear normal without clinical signs except a preputial discharge associated with small nodules on the preputial and penile membranes in the early stage of infection [1, 2]. Nevertheless, it can carry the parasite in the preputium with some concentration in the fornix and around the glans penis for an extended period of time, very likely for life [1, 2]. Therefore, a *T. foetus*-positive bull is always a major, if not the solely source of infection for a herd of cows served with natural mating. In contrast, a female cattle experiences vaginitis, endometritis, early abortion and transient or even permanent infertility after infection [3]. A cow or heifer may also develop a short-lived immunity and becomes pregnant again in the same breeding season with a delay, causing an extended and much longer calf season in a *T. foetus*-positive cattle herd [4].

*Tritrichomonas foetus* and bovine trichomonosis it causes in cattle have been found or presume to be worldwide on all continents except the Antarctica [5–8]. Bovine trichomonosis was first discovered in the US cattle in Philadelphia, Pennsylvania in 1932. Since then it has been found in many US states including Alabama, California, Colorado, Florida, Idaho, Kansas, Missouri, Montana, Nebraska, Nevada, New Mexico, Oklahoma, South Dakota, Utah and Wyoming [7]. Recently added to the list include Arkansas, Georgia, Hawaii, Illinois, Iowa, Kentucky, Louisiana, Mississippi, Oregon, Tennessee, Texas and Virginia [9–12]. Due to its capacity of causing great economic losses, which is briefly addressed below, 33 US states have rules on bovine trichomonosis currently in effect [12], mainly testing and culling *T. foetus*-positive bulls as well as prohibiting import of positive ones. These rules aim to control and eventually eradicate bovine trichomonosis.

## Economic loss

Accurately calculating the economic losses caused by bovine trichomonosis on both individual cattle producers and cattle industry as a whole is very challenging if not impossible. Economic losses caused by bovine trichomonosis mainly consist of diminished financial gains due to (1) financial costs of testing bulls and veterinary expenses, culling and replacing *T. foetus*-infected bulls and open cows; and (2) lower calf crop as a result of fewer calves born and lighter calf bodyweight.

### Bull testing and veterinary expenses

Factors that affect the cost for testing bulls include, but are not limited to, farm size in numbers of cows, bull to cow ratio, distance to veterinarian service and numbers of test performed. An estimate for bull-test cost per cow was made based on a survey of veterinarians in Colorado State is showed in Table 1. The estimated average cost for bull testing ranged from $1.20 to $5.68 per cow depending upon how far a farm was away from a veterinarian or whether bulls were hauled into a clinic [13]. The current fee schedule of Wyoming State Veterinary Laboratory (WSVL) for *T. foetus* test is $8.00/each for 1–2 samples or $6.00/each for ≥ 3 samples using culturing; $30.00/each for 1–9 samples or $25.00/each for ≥ 10 samples using PCR [14]. Using the same bull:cow ratio of 1:20 as in Table 1, the current average cost of bull testing in Wyoming is $0.40–1.50, $0.30–1.50, $0.30–1.25, and $0.30–1.25 per cow for a size of farm with 40, 100, 200 or 400 cows, respectively. Of course, an additional cost for veterinarian traveling and sample-taking needs to be added up to it.

**Table 1:** Estimated average cost in US dollars for bull testing in Colorado State in 2008*

Herd size-# of cows	“In clinic” (Total cost)	10 miles (Total cost)	75 miles (Total cost)
40	1.50 (60)	2.50 (100)	5.68 (227)
100	1.35 (135)	1.65 (165)	2.93 (293)
200	1.25 (250)	1.33 (266)	2.12 (424)
400	1.25 (500)	1.20 (480)	1.79 (716)

^*^The estimate was based on a bull to cow ratio of 1:20 with a farm 10 or 75 miles away from veterinarians or bulls being hauled to a veterinary clinic designated as “In clinic”. The cost was for one test of all bulls. The data are from [13].

### Direct losses

In Wyoming it was estimated that a 20% reduction in calf crop due to bovine trichomonosis in a 100-cow herd in 2011 could cause $20,000 in annual losses [15]. Further a computer modeling was adapted using 400 cows with 5 bulls where one or two bulls were positive with *T. foetus*. The model showed that (1) 14–50% reduction in annual calf crop; (2) 5–12% reduction in sucking/growing period; (3) 4–10% reduction in the bodyweight of calf crop at weaning; (4) 4–10% reduction in monetary return per calf born and (5) 5–35% reduction in in monetary return per cow [16].

## Wyoming’s long march to control and eradication of bovine trichomonosis

Wyoming started its journey for control and eradication of bovine trichomonosis in the year 2000 when it started a state rule called “Chapter 15” that mandates testing all bulls grazing on open/public allotments or being traded or leased for reproduction and culling *T. foetus*-positive bulls as well as prohibiting import of *T. foetus*-positive bulls. At the same time the rule listed bovine trichomonosis a state notifiable disease. The disease had been known in the state since 1970s, and the WSVL performed only hundreds of tests on bovine samples for *T. foetus* annually prior to 2000. Since then the numbers of samples tested annually have exponentially increased to thousands. The author had served as the WSVL Parasitology Section head between 2008 and 2013. A few specific aspects are worth discussion for the Wyoming’s journey.

### Risk factors for bovine trichomonosis

A statewide survey of all cattle producers in Wyoming was carried out in 2011 by the author and colleagues using a questionnaire. Twenty-five variables were included in the questionnaire for their possible association with the positive status among the *T. foetus*-infected cattle herds. These were on producers (educational level, annual family income and knowledge of bovine trichomonosis), herds (geographic area, bull number, bull breed, bull mean age, primary bull source, bulls purchased annually, co-mingling, breeding method, cow number, open-cow rate, pregnancy testing, vaccination, vaccine brand, vaccination frequency and vaccination season) and allotments (type, fences, frequency in fence checking, broken fence, time needed to fix a fence, positive herd(s) in neighborhood). An overall response rate was 23.4% (1288/5498). Risk factors identified and their odds ratio (OR) were: neighboring a positive herd(s) (OR = 18.3, 95% CL 4.1–81.1, *P* = 0.0003), grazing on public allotments (OR = 2.9, 95% CL 0.7–12.1, *P* = 0.003), commingling with other herd (OR > 999.9, *P* = 0.026, 95% CL > 999.9) and elapsed time taken to fix a broken fence(s) a week or longer (OR = 4.3, *P* = 0.078, 95% CL 0.9–20.2) [17].

### Unpopularity of artificial insemination (AI)

In the USA, cattle producers are voluntary in performing AI among their cattle herds. Not a single US state has a decree that demands mandatory AI. In the above-mentioned questionnaire cattle producers were also surveyed for breeding methods in their cattle herds. Overwhelming 80.8% of cattle herds were by live bull service whereas only 2.1% was by AI in the year 2010. Furthermore, 56.1% of producers in Wyoming would not consider AI and only 36.7% would in the future [17]. Therefore, AI, the method having proved effective in European Union countries, would have very marginal effect in Wyoming State for control and eradication of bovine trichomonosis due to its unpopularity among cattle farmers.

### *T. foetus* strain

Clinical isolates of *T. foetus* from Wyoming positive bulls were cultured and their identity was investigated by sequencing partial small and large subunit rRNA, the internal transcribed spacer 1 and 2, and 5S rRNA fragments followed by a phylogenetic analysis. All 24 isolates had identical sequences and were confirmed as bovine strain [18]. It is worthy of mentioning an interesting case just published this past April. The preputial fluid of a Polish bull was microscopically and PCR positive for *T. foetus* after culture. Nevertheless, DNA sequence of the PCR amplicon showed 99.62% identity with *Honigbergiella* sp [19], which may be present in cattle feces [20]. Therefore, it is important to confirm that a PCR amplicon truly belongs to *T. foetus* by DNA sequencing, which is especially important in the *T. foetus*-free or eradicated regions/countries for maintaining such a status.

### Testing cows and aborted fetuses

In addition to routine testing bull for *T. foetus*, the WSVL had been testing cows with abortion and aborted fetuses for the pathogen. Both cell culture and conventional PCR were employed. In a span of 11 years from 2000 to 2010, 9.7% (9/93) such cows were tested positive, and 4.5% (1/22) of aborted fetuses were similarly tested positive. These cows may play a role in maintaining endemicity of bovine trichomonosis in the corresponding herds [21] based on the fact that some cows carry the protozoan for an extended period of time, i.e., up to 300 days post-breeding [4, 22]. Therefore, it is necessary to test cows with a history of abortion and open cows, especially in suspected herds for the protozoan.

### Bull testing

It was not until 1990s that the WSVL provided routine test for *T. foetus* although it started the test in 1970s. Wyoming state rules on bovine trichomonosis (Chapter 15) has been into effect since 2000 with the Wyoming Livestock Board’s enforcing them. State laws mandate bulls grazing on open/public allotments or being traded or leased for the reproductive purpose be tested prior to breeding or change of ownership. They are usually tested in the winter season prior to their being put on pasture the next spring. In 1999, the year before state’s rules of the Chapter 15 came into effect, the statewide prevalence of individual cattle was 2.69%. There was a slow but steady decline since then. In 2008, the statewide prevalence was reduced to 0.62%, a 77% reduction in perveance in nine years. However, the statewide prevalence reached 1.29% in 2009, a 100% increase than the previous year, before it set back to 0.21% in 2010. Based on the data, a linear regression was established as: Prevalence F(x) = 261.020047–0.129685*Year(x) (r = 0.717; *P* = 0.009) [7]. The linear model predicates that by 2013 Wyoming would have no positive cases of *T. foetus* in bulls if the model were allowed to be used beyond the range of years of its data collection. This had not happened. In next five years from 2011 to 2015, the statewide prevalence was between 0.12 and 0.5% with over 10 000 bulls tested annually. In 2016, the statewide prevalence dropped to 0.03% with only three positive bulls. Further in 2017, 2018 and 2019, three years in a row, not a single *T. foetus*-positive bull was found throughout the entire state (Table 2). It is worth a short discussion what caused the resurgence of the statewide prevalence of individual cattle observed in 2009. The statewide prevalence of cattle herds in 2009 was 2.16%, lower than 2.55% and 3.21% in the previous two years of lower statewide prevalence of individual cattle [7]. Therefore, it is plausible that an increase of the positive individual cattle among positive herds was the main, and possibly the solely reason for this resurgence. A similar resurgence in the future is very unlikely since now all herds throughout the entire state are *T. foetus*-free. It is safe to conclude that Wyoming has finally reached its goal of total control and eradication of bovine trichomonosis statewide 20 years after it started a long march in 2000.

**Table 2:** ***Tritrichomonas foetus*** **prevalence of Wyoming individual bulls**

Year	No tested	No positive	Prevalence (%)	References
1997	433	5	1.15	[7]
1998	920	18	1.96	[7]
1999	1525	41	2.69	[7]
2000	4880	71	1.45	[7]
2001	6025	78	1.29	[7]
2002	6515	71	1.09	[7]
2003	6855	43	0.63	[7]
2004	7515	44	0.59	[7]
2005	7450	79	1.06	[7]
2006	7270	57	0.78	[7]
2007	7080	50	0.71	[7]
2008	7275	45	0.62	[7]
2009	7597	98	1.29	[7]
2010	8222	17	0.21	[7]
2011^†^	10,301	18	0.17	[25]
2012	10,172	51	0.50	[25]
2013	10,142	25	0.25	[25]
2014	10,168	12	0.12	[25]
2015	10,054	14	0.14	[25]
2016	10,826	3	0.03	[25]
2017	11,369	0	0	[25]
2018	10,680	0	0	[25]
2019	9920	0	0	#

^†^Starting the year 2011, the data were from July 1 of the year to June 30 of the next one instead of the calendar year of January 1 to December 31 for the data presented from 1997 to 2010.

^#^Personal communication with permission of Dr Jim Logan and Dr Douglas Leinart of Wyoming Livestock Board.

## Concluding remarks and prospects

Based on published and open-access data, this manuscript concludes that Wyoming State has eradicated bovine trichomonosis. This is achieved even though (1) AI was scarcely used by Wyoming cattle producers. (2) Very low percentage of bulls had been tested three times. The state must continuously enforce its rules since all its adjacent states are still endemic with bovine trichomonosis. In total 33 US states have similar state rules in effect [12]. These rules cannot and should not be abandoned until the entire USA eradicates the disease, which may still be many years to come.

**Figure 1:**
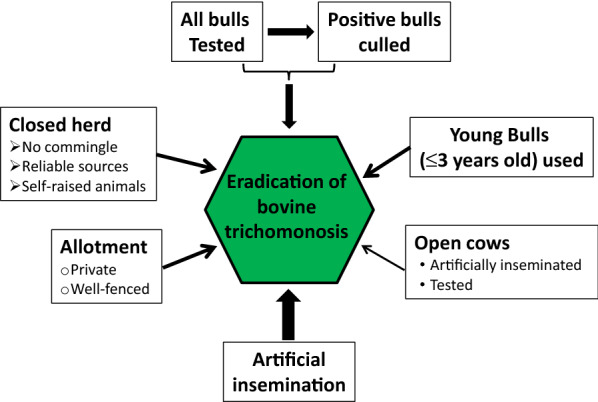
**Control measures for bovine trichomonosis**. Thickness of an arrow indicates importance of a measure for control and eradication of bovine trichomonosis. The thicker an arrow, the more effective it is. Adapted from references [17, 26].

Can this success be repeatedly achieved in other US states and rest of the world? To control the disease for minimizing economic losses regulations and rules/laws similar to those of Wyoming are in effect in other 33 US states [12]. To achieve eradication culling *T. foetus*-positive bulls is necessarily carried out without any reservation. This is also indirectly supported by the data generated from La Pampa, Argentine. La Pampa is an administrative body in Argentine like Wyoming is a state in the USA. It started a mandatory testing and culling program in 2008. The positive rate of bulls reduced to 1.02% in 2011 from 2.34% in 2008, a 56.4% decrease in only four years. However, the rate remained flat in the next four years between 2012 and 2015. Not culling positive bulls was to be blamed for this flat persistence of bovine trichomonosis [23]. In addition, three negative tests are recommended for ruling out of possible infections in high risk situations [24]. Collectively, strict enforcement of testing and culling *T. foetus*-positive bulls besides of AI will lead to total control and the final eradication of bovine trichomonosis. Further, its complement with several other measures such as keeping closed herds, using bulls of three years old or younger and well-fenced allotments will accelerate eradication (Figure 1).

## Abbreviations

AI: artificial insemination
OR: odds ratio
WSVL: Wyoming State Veterinary Laboratory
